# Functional Interaction of Cockroach Allergens and Mannose Receptor (CD206) in Human Circulating Fibrocytes

**DOI:** 10.1371/journal.pone.0064105

**Published:** 2013-05-29

**Authors:** Ying-Ming Tsai, Shih-Chang Hsu, Jian Zhang, Yu-Feng Zhou, Beverly Plunkett, Shau-Ku Huang, Pei-Song Gao

**Affiliations:** 1 Johns Hopkins Asthma and Allergy Center, Johns Hopkins University School of Medicine, Baltimore, Maryland, United States of America; 2 Department of Pulmonary and Critical Care Medicine, Kaohsiung Medical University, Kaohsiung, Taiwan; 3 Department of Respiratory Medicine, Xijing Hospital, Fourth Military Medical University, Xi’an, P.R. China; 4 National Health Research Institutes, Zhunan, Taiwan; Ludwig-Maximilians-University Munich, Germany

## Abstract

**Background:**

The innate pattern recognition C-type-lectin receptors (CLRs), including mannose receptor (MRC1; CD206), have been suggested to functionally interact with allergens and are critical in controlling immune response. Fibrocytes have been considered to play a role in allergic asthma. Here we sought to investigate the functional interaction of cockroach allergens with CD206 in fibrocytes.

**Methods:**

Profiling of N-linked glycans from natural purified cockroach allergen Bla g 2 was accomplished by MALDI-MS. The binding activity of cockroach allergens to CD206 was determined by solid-phase binding assays. Levels of CD206 expression on human fibrocytes and CD206 mediated signaling and cytokine production in Bla g 2 treated fibrocytes were determined.

**Results:**

Profiling of N-linked glycans from Bla g 2 revealed a predominance of small, mannose-terminated glycans with and without fucose. Significant binding of Bla g 2 to CD206 was observed, which was inhibited by yeast mannan (a known CD206 ligand), free mannose, and a blocking antibody (anti-hMR). Flow cytometric analyses of human fibrocytes (CD45^+^ and collagen-1^+^) showed selective expression of CD206 on fibrocytes. Functionally, a concentration-dependent uptake of FITC labeled Bla g 2 by fibrocytes was observed, but was significantly inhibited by anti-hMR. Bla g 2 can stimulate up-regulation of inflammatory cytokines including TNF-alpha and IL-6 and activation of nuclear factor kappa B (NF-kB/p65), p38 mitogen-activated protein kinase (p38), ERK, and JNK in cultured fibrocytes. This increased secretion of TNF-alpha and IL-6 and activation of NF-kB, ERK, and JNK was significantly inhibited by the addition of either mannan or mannose. Furthermore, Bla g 2 induced increase in TNF-alpha and IL-6 production was also inhibited by the use of NF-kB, ERK, and JNK inhibitors.

**Conclusion:**

These results provide evidence supporting the existence of a functional cockroach allergen-CD206 axis in human fibrocytes, suggesting a role for CD206 in regulating allergen induced allergic responses in asthma.

## Introduction

Asthma is the leading serious chronic illness of children in the US. Exposure to cockroach allergen in early life can lead to allergic sensitization and increase the risk of developing asthma [1], [2], [3], [4]. In inner-city populations, 60–80% of children with asthma are sensitized to cockroach [3], [5]. Recent studies in the New York City Neighborhood Asthma and Allergy Study (NAAS) have found that cockroach allergen (Bla g 2) was more prevalent in bed dust from homes in high asthma prevalence neighborhoods (HAPN) than that from low asthma prevalence neighborhoods (LAPN) [6]. These studies support the notion that cockroach exposure increases the risk of allergic sensitization, which in turn leads to the development of asthma. At present, reducing exposure is still the best option for alleviating potential cockroach induced asthma [7], highlighting the need to understand the mechanism of cockroach induced sensitization and to develop therapeutic strategies.

Complex allergens contain multiple innate immune-activating components, which trigger the activation of mucosal innate immune cells that subsequently promote Th2-polarized adaptive immune responses and IgE responsiveness in susceptible individuals [8], [9], [10], [11]. For instance, protease-activated receptor (PAR)-2, a receptor for cockroach-derived protease, has been shown to mediate activation of airway epithelial cells [11], [12] and development of allergic diseases [13], [14], [15]. German cockroach frass has been shown to directly affect neutrophil cytokine production via Toll-like receptor (TLR)-2, suggesting cockroach allergen may contain a TLR-2 ligand [16], [17]. Both Bla g 1 and Bla g 2 are major purified German cockroach allergens as determined by the prevalence of IgE-mediated responses to them (30–50% and 60%, respectively) [18]. Bla g 2 is an especially potent antigen, inducing IgE antibody responses at very low doses of exposure (0.33 ug/g) [4] [19], [20]. Although Bla g 2 shares sequence homology with the aspartic proteinase family of proteolytic enzymes, it lacks proteolytic activity in a standard milk-clotting assay using casein as a substrate [21], [22]. These findings suggest that enzymatically inactive factors, not dependent on enzymatic activity, play a role in cockroach induced immunological response.

CD206 (MRC1) encodes the mannose receptor C-type lectin (MR), a cell surface protein that belongs to a family of C-type lectin receptors (CLRs). CLRs are crucial in recognition of complex glycan structures on various pathogens and have evolved to facilitate the endocytosis and presentation of pathogens [23], [24], [25], [26]. CD206 contains 8 C-type lectin-like domains (CTLD) and acts as a phagocytic receptor for bacteria, fungi and other pathogens [27]. CTLD4, 5, and -7 are required for high affinity binding and endocytosis of multivalent glycoconjugates [28]. Recent studies have demonstrated that immature monocyte-derived dendritic cells (MMDCs), bone-marrow derived DCs (BMDCs) and macrophages via CD206 mediate the uptake of diverse native allergens and subsequent allergic sensitization as well as allergen induced T cell polarization [29], [30]. Furthermore, MMDCs from allergic patients have been shown to be more efficient in antigen uptake via CD206 than those from healthy individuals [31]. Specifically, CD206 on MMDCs mediates Bla g 2 uptake via the C-type lectin-like carbohydrate recognition domains (CTLD)4–7 [30]. A recent study has demonstrated that CD206 is expressed in fibrocytes [32].

Fibrocytes, a unique form of bone marrow-derived mesenchymal cell progenitors, comprise a minor component of the circulating pool of leukocytes (<1%) and express markers of both hematopoietic cells (CD34, CD43, CD45, MHC class II) and stromal cells (collagen I and III) [32], [33], [34], [35]. These cells are distinguished from monocytes, macrophages and DCs by their cell surface markers, morphology, and growth properties [36]. Increased numbers of local and/or circulating fibrocytes were observed in patients with asthma [37]. Importantly, the increased circulating fibrocytes were correlated with persistent airway flow obstruction in patients with asthma, and fibrocytes from patients with chronic obstructive asthma have a greater capacity for proliferation and differentiation, suggesting a possible role of fibrocytes in airway inflammation and remodeling [36], [37]. Furthermore, fibrocytes, like DCs, function as antigen-presenting cells (APCs) capable of priming naïve T cells in situ [34]. It has been suggested that DCs may be important in priming and activating the T cells in the secondary lymphoid tissues and at the site of tissue mucosa, while fibrocytes may play a role in promoting DC activation and in modulating the DC mediated antigen-specific T cell responses [36], [38]. In addition, fibrocytes can produce extracellular matrix (ECM), differentiate into fibroblasts, and play an important role in airway remodeling [36] [39], [40]. However, few studies have focused on the assessment of fibrocyte function, such as antigen binding, uptake and modulation of allergen induced immune responses.

In the present study, we have performed the profiling of N-linked glycans from purified natural Bla g 2, and investigated the functional interaction between Bla g 2 and CD206 in antigen binding and antigen recognition and downstream immune responses by fibrocytes. The cellular mechanisms triggered by the interaction of Bla g 2 and CD206 were also investigated.

## Materials and Methods

### Profiling of N-linked Glycans from Natural Purified Bla g 2 Using Mass Spectrometry

The glycan preparation from Bla g 2 glycoprotein(INDOOR Biotechnologies, Charlotesville, VA) and profiling of glycan analysis by mass spectrometry was performed at the Core for Glycan Analysis at Georgia Institute of Technology, GA, USA, and detailed methods were summarized in Materials and Methods in the Online Repository. N-linked glycans are those released from resulting peptides/glycopeptides via digestion with peptide N-glycosidase F (PNGase F).

### Binding Assays of Cockroach Allergens and Soluble CD206

Interaction between CD206 and cockroach allergen was detected by binding assays as previously described [41]. Serial dilution of allergens including cockroach extract (CRE, 10 ug/ml, GREER, Lenoir, NC), purified Bla g 2 (10 µg/ml), mannosylated BSA with 51 mannosides coupled to BSA (Man_51_-BSA/Man-BSA, 10 µg/ml; as a positive control) [41], [42] and BSA (10 µg/ml, as a negative control) were coated in 0.05 M carbonate-bicarbonate buffer (pH 9.6) onto assay plates by overnight incubation at 4°C. The plates were then incubated with 0.5 ug/ml of soluble CD206 containing the Fc portion of human IgG1 (R&D Systems, Minneapolis, MN) at room temperature for 2 h. The binding was detected by incubation with peroxidase-conjugated goat anti-human IgG Fc antibody (Pierce, Rockford, IL) for 1 h at room temperature and by the addition of a substrate, tetramethylbenzidine (R&D Systems). The relative binding activity was expressed as optical density (OD) for each test antigen after subtracting the values from the background. To confirm the binding specificity, inhibition assays were performed with serial dilution of anti-hMR blocking antibody (3.0 µg/ml, clone 19.2, BD Pharmingen, Mississauga, Ontario), mannan (3.0 mg/ml, Sigma), a CD206 ligand [43], [44], and mannose (100.0 µg/ml, Sigma, Oakville, Ontario) added and incubated for 1 h before the allergens were added to the plates for binding assays.

### C-type Lectin Receptor (CLR) Expression in Fibrocytes

Circulating fibrocytes were isolated according to Young's protocol [45]. Study protocols were approved by the Institutional Review Board at the Johns Hopkins University School of Medicine. Written informed consents were obtained from each of the study subjects.

PBMCs (6–8×10^7^ cells) were isolated from 50 ml blood of cockroach allergic subjects using Ficoll-Paque PLUS (GE Healthcare, Pittsburgh, PA) according to the manufacturer’s instructions, and then cultured in fibrocyte culture medium (2×10^6^ cells/ml) (high glucose DMEM+20% FBS) [45]. After 48 hours, non-adherent cells were removed. After 14 days, adherent cells were washed with HBSS and harvested using trypsin digestion. Fibrocyte purity and characterization were determined by flow cytometry analysis with CD45 (clone 2D1) CD16 (clone 3G8), CD32(clone 3D3), and CD23 (clone M-L233) from BD Biosciences Pharmingen, polyclonal COI I, PM-2K (clone PM-2K), CD209 (clone 5D7), and FcεRI (clone 9E1) from Abcam (Cambridge, MA). For CD206 intracellular expression analysis, 2×10^5^ fibrocytes were fixed with Fix/Perm Buffer (BD Biosciences) for 30 minutes at 4°C. Then cells were permeabilized with permeabilization buffer (BD Biosciences) for 5 minutes, and staining was performed with the fluorescence-labeled primary antibodies, anti-human CD206 (Abcam), and isotype-matched controls for 30 min at 4°C. Cytometric data were analyzed by using FACS Calibur (Becton-Dickinson, San Jose, CA, USA). The resultant data were analyzed using FlowJo (Tree Star, Ashland, OR). The images were also captured using a Zeiss LSM 510 META confocal microscope (Carl Zeiss, Thornwood, NY).

### CD206 Mediated Cockroach Allergen Uptake by Fibrocytes

For allergen uptake assays, the purified native Bla g 2 was labeled with Lightning-Link FITC Antibody Labelling Kit (Novus, UK). Different doses of FITC labeled purified Bla g 2 were co-incubated with the cultured fibrocytes (2×10^5^) for 1 h at 37°C. Bla g 2 uptake was detected by flow cytometry, and co-localization between FITC-labeled Bla g 2 and CD206 in fibrocytes was detected by immunofluorescence staining. To confirm the allergen uptake specificity, inhibition assays were performed with anti-hMR (clone 19.2, BD Pharmingen) or an isotype IgG2a control Ab pre-treatment for 1 h.

### ELISA of Cytokines from Bla g 2 Treated Fibrocytes

Cockroach allergen induced fibrocyte activation was determined by IL-6 and TNF-α secretion in supernatants of cultured fibrocytes when exposed to Bla g 2 (10 µg/ml). The levels of IL-6 and TNF-α were measured by ELISA (BD Biosciences, San Jose, CA, USA), according to the manufacturer’s instructions. To examine the involvement of CD206 in cytokine production, we pretreated the fibrocytes for 1 h with different doses of mannan (0.5–2 mg/ml) and mannose (1–25 mg/ml) before exposure to Bla g 2 (10 µg/ml). In some cases, the cells were also pre-treated with inhibitors for NF- kB (BAY-11-7082), p38 (SB 203580), ERK (PD98059), and JNK (SP600125) (Calbiochem, Rockland, Massachusetts, USA) 5 µM for each inhibitor for 1 h.

### Western Blot Analysis of CD206 Mediated Signaling

To analyze the Bla g 2-induced signaling events, we treated fibrocytes with Bla g 2 (10 µg/ml) for various times (0, 15, 30, 60, 120 min). To further examine the modulatory effects of CD206, cultured fibrocytes were pre-treated with mannan (2.0 mg/ml) for 1 h before Bla g 2 treatment. After stimulation, the total cellular extracts (30 µg) were subjected to 10% to 13% SDS-polyacrylamide gel electrophoresis and probed with antibodies against various kinases and phosphorylated kinases as previously described [41]. The antibodies used included anti-p65, anti-phospho-p65, anti-p38, anti-phospho-p38, anti-ERK1/2, anti-phospho-ERK1/2, and anti-JNK and anti-phospho-JNK (Cell Signaling Technology Inc, USA). The relative levels of phosphorylated proteins were quantified by ImageJ (National Institutes of Health, USA) for the densitometric analysis of the band intensities and normalized to those of β-actin.

### Statistical Analysis

Statistical significance for normally distributed samples was analyzed using an unpaired Student’s *t*-test by using GraphPad Prism version 5.1 software (GraphPad Software, La Jolla, CA). Differences with *P*<0.05 were considered statistically significant.

## Results

### Bla g 2 Contains Mannose-terminated Glycans with and without Fucose

**Figure 1:**
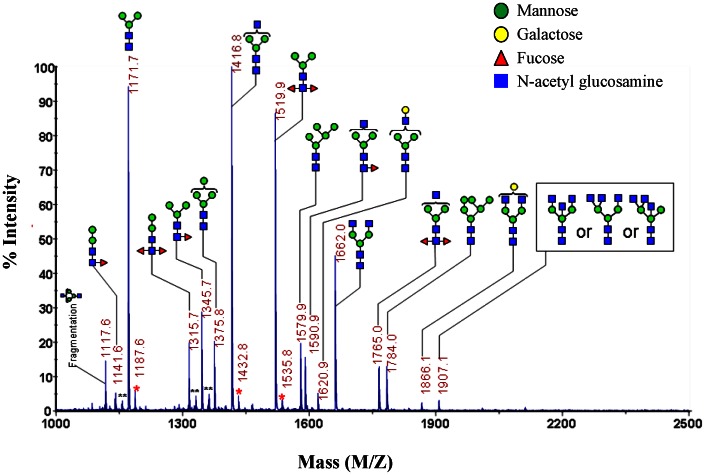
Full MALDI-TOF mass spectrum of permethylated N-linked glycans from *Bla g 2*. N-linked glycans were prepared from purified natural Bla g2 glycoprotein and analyzed by MALDI-TOF mass spectrum. Glycan compositions were assigned by comparison of measured m/z values with m/z values calculated based on putative composition of permethylated glycans. X axis: mass to charge ratio (m/z). Green circle: Mannose; yellow circle: galactose; red triangle: fucose; and blue square: N-acetyl glucosamine. Y axis: signal intensity of the ions. **is undermethylated glycans. *is unknown peaks.

We have performed the profiling of N-linked glycans isolated from natural purified cockroach allergen Bla g 2 by MALDI-TOF mass spectrum (MALDI-MS). N-linked glycans were enzymatically cleaved from Bla g 2, and permethylated. Native and permethylated N-glycans were further analyzed by MALDI-MS. The proposed compositions of released N-linked glycans from Bla g 2 are presented in **Fig. 1** and **Table S1** in the Online Repository. Glycan compositions were assigned by comparison of measured m/z values with m/z values calculated based on putative composition of native glycans using the Functional Glycomics glycan database (www.functionalglycomics.org) and SimGlycan software (Premier Biosoft, Palo Alto, CA) [46]. We found that natural purified cockroach allergen Bla g 2 contains putative structures that have a significant amount of glycans, and many of these terminate with mannose. The most intense N-linked glycan compositions in Bla g 2 are Man3GlcNAc2 (*m/z* 1171.7), Man3GlcNAc3 (*m/z* 1416.8), and Man3Fuc2GlcNAc2 (*m/z* 1519.9).

### Binding of Cockroach Allergen to Soluble Human CD206

**Figure 2:**
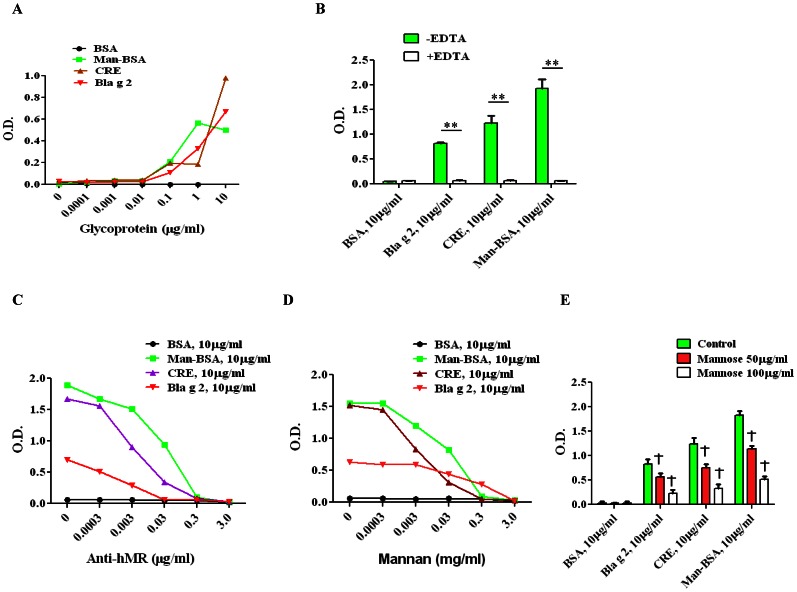
Binding assays of cockroach allergens and soluble CD206. (**A**) Serial dilution of 10 µg/ml of allergens including cockroach extract (CRE), purified Bla g 2, and positive (Man-BSA) and negative (BSA) controls were coated onto assay plates, and then probed with soluble CD206. (**B–E**) Inhibition assays were performed with or without 5 mM EDTA (**B**), and with serial dilution of anti-hMR (**C**, 3.0 µg/ml), mannan (**D**, 3 mg/ml), and mannose (**E**, 100 µg/ml) added and incubated for 1 h before the allergens were added to the plates for binding. The relative binding activity was expressed as optical density (OD). Data are mean ODs of three individual experiments. **P<0.01 (**B**), ^†^ *P*<0.01 (**E**, compared to medium control).

To determine if cockroach allergen that contains mannose can directly bind CD206, we analyzed the binding of both German cockroach extract and purified Bla g 2 to soluble human CD206 using ELISA for the detection of Bla g 2-CD206 binding. In this analysis, BSA and Man-BSA were used as a negative and positive control, respectively. The results showed that similar to that found for positive control (Man-BSA), significant binding to CD206 was found for both cockroach extracts (CRE) and Bla g 2, but not BSA (**Fig. 2A**). The binding was abrogated by the addition of 5 mM EDTA (**Fig. 2B**), suggesting that the binding was Ca^++^-dependent. Further, the allergen binding abilities were significantly inhibited by using CD206 blocking antibody anti-hMR (**Fig. 2C**), mannan (**Fig. 2D**) and mannose (**Fig. 2E**) in a concentration-dependent manner.

### Characterization of Human Circulating Fibrocytes

**Figure 3:**
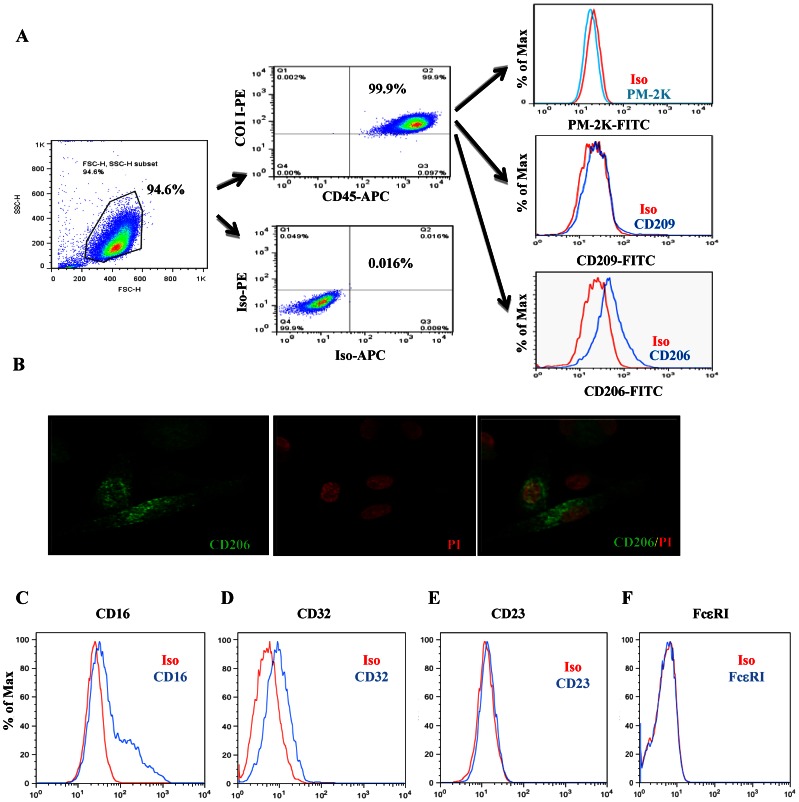
Characterization of human circulating fibrocytes. Co-expression of two fibrocyte markers CD45 and COI I in cultured human fibrocytes. (**A**) ∼95% of cells showed co-expression of CD45^+^ and COI I^+^ as determined by flow cytometry; those resting cells are negatively stained for macrophage marker PM-2K or DC marker CD209, but positive for CD206 in fibrocytes. (**B**) CD206 expression (green) in fibrocytes was further confirmed by confocal microscopy. Nuclear DNA is stained with propidium iodide (PI) (original magnification x60). Markers CD16 (**C**), CD32 (**D**), CD23 (**E**), and FcεRI (**F**) were constitutively expressed on cultured fibrocytes detected by flow cytometry. Figures are representative of three independent experiments.

To determine the role of fibrocytes in cockroach allergen induced immune responses, we used cultured fibrocytes derived from human peripheral blood mononuclear cells (PBMCs) [45]. We have assessed the purity of these fibrocytes by CD45 and COI I, and found that ∼95% of cultured cells co-expressed CD45 and COI I (**Fig. 3A**). We have also assessed the specificity of these cells by using PM-2K (marker for macrophages) and CD209 (marker for DCs), and found that none of the cells expressed PM-2K and CD209. CD206 was constitutively expressed in cultured fibrocytes by using flow cytometry. CD206 expression on fibrocytes was further confirmed by confocal microscopy (**Fig. 3B**), which also showed intracellular expression pattern. Furthermore, we found that fibrocytes express both CD16 (FcγRIII**,** **Fig. 3C**) and CD32 (FcγRII**,** **Fig. 3D**), but neither IgE low affinity receptor (CD23, **Fig. 3E**) nor high affinity receptor (FCεRI, **Fig. 3F**).

### CD206 Mediates Cockroach Allergen Uptake by Fibrocytes

**Figure 4:**
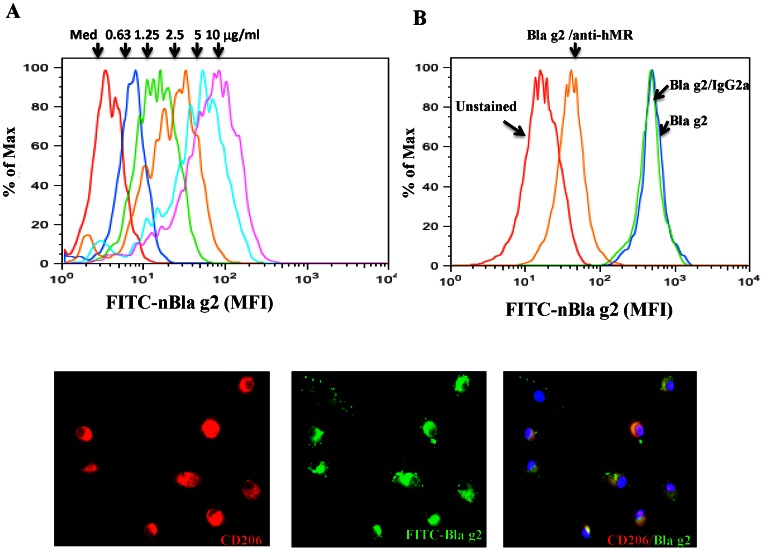
CD206 mediates Bla g 2 uptake by fibrocytes. (**A**) Different doses of the FITC labeled purified Bla g 2 were co-incubated with fibrocytes for 1 h at 37°C, Bla g 2 uptake was detected by flow cytometry (Colors represent different doses as indicated). (**B**) The Bla g 2 uptake (blue line) was significantly inhibited when cells were pre-incubated with CD206-blocking anti-hMR (orange line) for 1 h at 37°C, but not with isotype (IgG2a, green line). (**C**) Co-localized Bla g 2 and CD206 in fibrocytes was detected by confocol microscopy. Nuclear DNA was stained by DAPI (original magnification x40). Figures are representative of three independent experiments.

To determine the involvement of CD206 in cockroach allergen uptake, we investigated Bla g 2 uptake by fibrocytes in the absence or presence of CD206-blocking antibody anti-hMR. The ability of Bla g 2 uptake was determined by flow cytometry. We found that the FITC-labeled Bla g 2 was taken up by fibrocytes in a concentration-dependent manner (**Fig. 4A**), which was significantly inhibited by anti-hMR, but not by isotype control IgG2a (**Fig. 4B**), suggesting that CD206 might be involved in the uptake of Bla g 2 by fibrocytes. Furthermore, Bla g 2 and CD206 were found to be co-localized in fibrocytes when FITC-labeled Bla g 2 was co-cultured with fibrocytes and stained with antibody against CD206 (**Fig. 4C**).

### CD206 Mediated Cockroach Allergen Induced Cytokine Production

**Figure 5:**
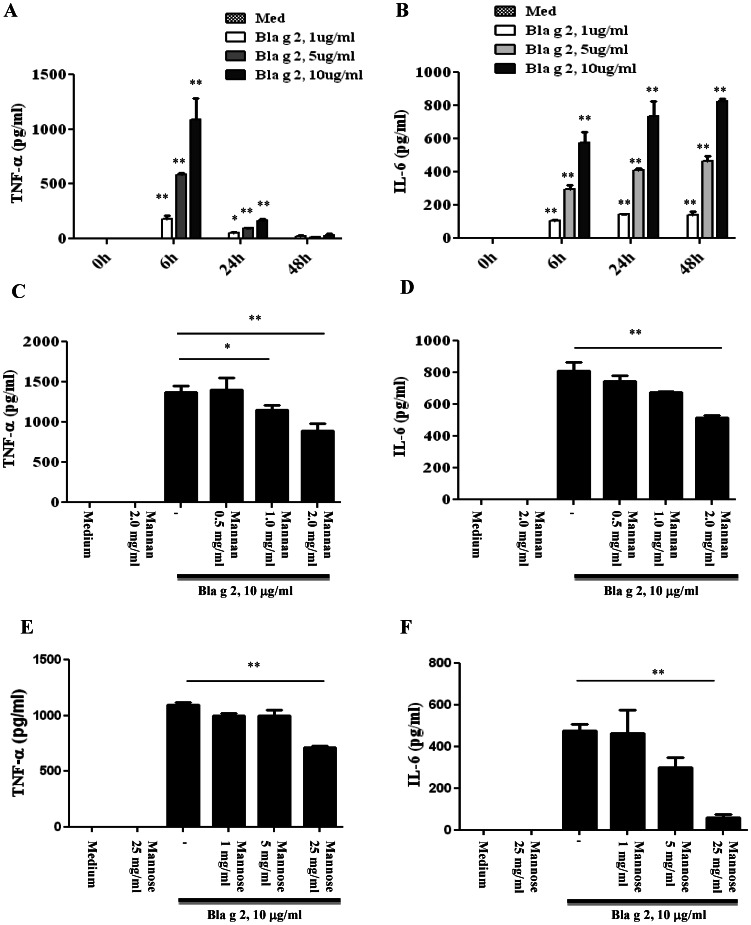
Bla g 2 induced TNF-α and IL6 secretion by fibrocytes was partially through CD206. (**A–B**) Different doses of Bla g 2 induced cytokine production at different times in supernatants of cultured fibrocytes including TNF-α (**A**) and IL6 (**B**) were detected using ELISA kits. (**C–F**) Fibrocytes were pretreated with either mannan (3 mg/ml) or mannose (25mg/ml) before Bla g 2 treatment (10 µg/ml), levels of fibrocyte secreted TNF-α (**C, E**) and IL6 (**D, F**) were detected. Data are mean ± SD of three individual experiments. *P<0.05, **P<0.01.

We measured levels of TNF-α and IL6 in supernatants of cultured fibrocytes when exposed to purified low-endotoxin natural Bla g 2 (<0.03 EU/µg protein). Significantly increased production was observed for TNF-α at 6 h (**Fig. 5A**) and IL-6 at 24 h (**Fig. 5B**) in a concentration- dependent manner when fibrocytes were exposed to Bla g 2 (10 µg/ml), compared to resting cells. To further test the importance of CD206 in Bla g 2 induced cytokine secretion, fibrocytes were pre-treated with different doses of mannan (0.5–2 mg/ml) and mannose (1–25 mg/ml) for 1 h before Bla g 2 treatment. The increased TNF-α and IL-6 secretions were significantly inhibited when fibrocytes were pre-treated with mannan at 2 mg/ml (**Fig. 5C, D**) and mannose at 25mg/ml (**Fig. 5E, F**). It is noted that neither mannan nor mannose alone stimulated TNF-α and IL-6 production even when the maximum dose in this study was used.

### Bla g 2-induced Signaling Events in Fibrocytes

**Figure 6:**
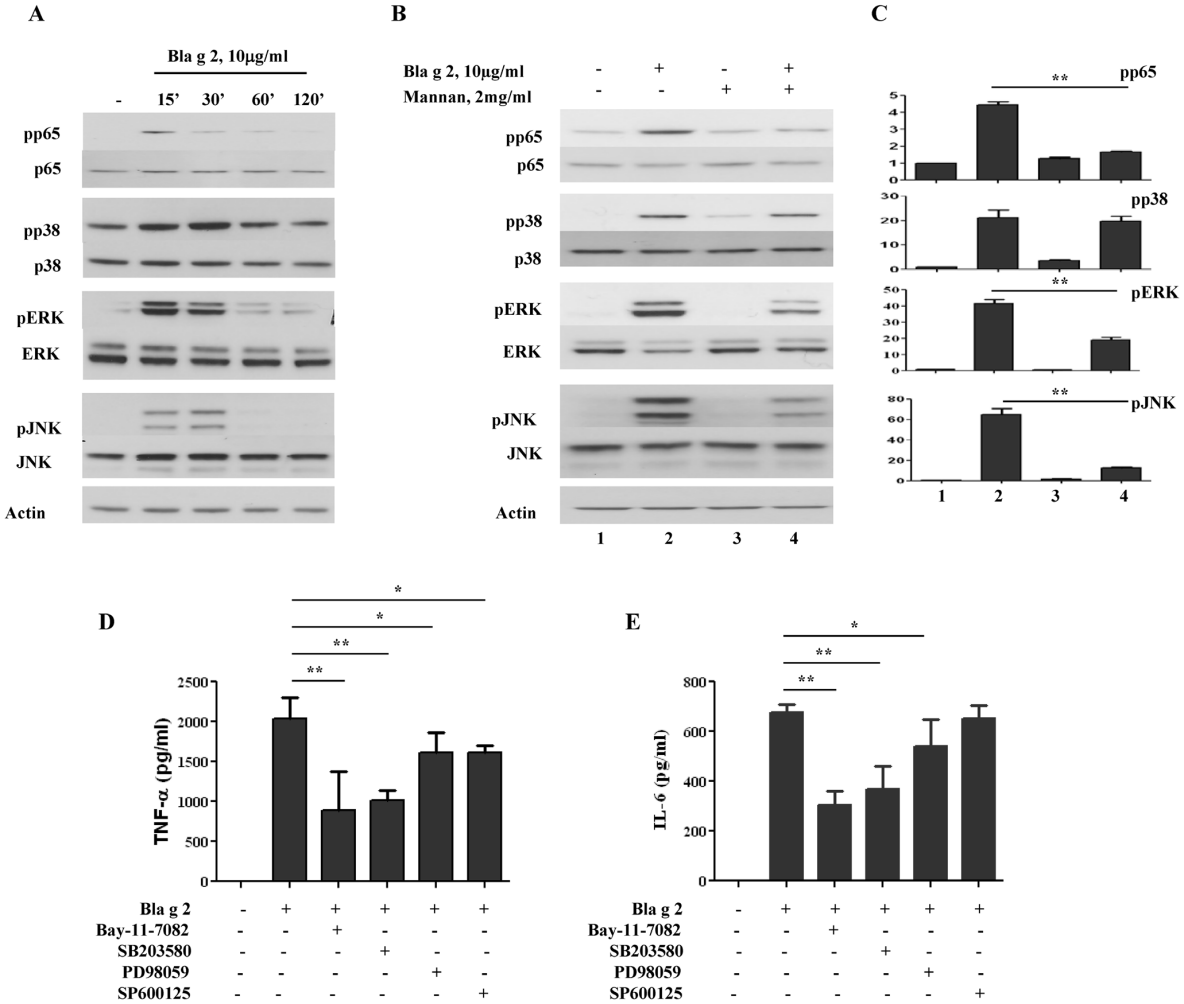
Bla g 2 induced NF-kB, ERK, and JNK activation through CD206 in fibrocytes. (**A**) Fibrocytes were treated with Bla g2 (10 µg/ml) at indicated times, and cell lysates were harvested for the detection of phospho-p38 MARP, p65, ERK, and JNK. (**B–C**) Fibrocytes were treated with Bla g 2 (10 µg/ml) or pretreated with mannan (2 mg/ml) for 1 h and cell lysates were harvested at 30 min for Western blotting with the indicated antibodies (**B**). The levels of phosphorylated proteins were quantified and normalized to those of β-actin (n = 3), and fold changes were generated when fibrocyte treated groups with either Bla g 2 or mannan was compared to the group without treatment (**C**). (**D–E**) Fibrocytes were treated with Bla g 2 (10 µg/ml) or pretreated with 5 µM inhibitors for NF-kB (Bay-11-7082), p38 (SB203580), ERK (PD98059), and JNK (SP600125) for 1 h at 37°C before Bla g 2 treatment. Levels of fibrocyte secreted TNF-α at 6 h (**D**) and IL6 at 24 h (**E**) in supernatant were detected using ELISA. Data are mean ± SD of three individual experiments. *P<0.05, **P<0.01.

To delineate the underlying mechanisms of Bla g 2-induced inflammatory cytokines in fibrocytes, the signaling events in the Bla g 2-CD206 axis were examined. Specifically, we analyzed the activation of NF-kB (p65), p38 mitogen-activated protein kinase (MAPK), c-Jun N-terminal kinase (JNK), and ERK signaling in fibrocytes in response to Bla g 2. After exposure to Bla g 2 (10 µg/ml), fibrocytes were activated at various times (15 min to 1 h, **Fig. 6A** **)** as determined by the levels of phosphorylated p65, p38, ERK, and JNK. The findings suggest that activation of those signaling molecules may be critical in cockroach allergen induced immune responses. To assess whether CD206 is involved in the activation of those signaling events, the mannan (2 mg/ml) was used to pre-treat the fibrocytes for 1 h at 37°C. We found that the allergen induced increased activation was suppressed by mannan (**Fig. 6B, 6C** **)**, particularly, that of p65, ERK, and JNK, suggesting that CD206 may play a role in determining the stimulatory activity of allergens in fibrocytes. Furthermore, Bla g 2 induced TNF-α (**Fig. 6D** **)** and IL-6 **(** **Fig. 6E** **)** secretions were significantly inhibited by the inhibitors for p65, p38, and ERK. However, the JNK inhibitor showed inhibition only for TNF-α, but not IL-6.

## Discussion

Cockroach exposure and sensitization poses a significant risk for developing asthma [4]. However, the regulatory mechanism is not clear and the causal relationship between allergen exposure and allergic sensitization and asthma remains to be established. In this study, we have discovered a novel regulatory pathway involving the functional interaction of cockroach allergen and CD206 in fibrocytes. In particular, we have demonstrated that cockroach allergen contains a CD206 natural ligand and functionally interacts with CD206 in fibrocytes, leading to the induction of proinflammatory cytokine production and activation of NF-kB, ERK, and JNK.

Cockroach extract contains protease, and studies on PAR-2 deficient mice have demonstrated an important role for PAR-2 in mediating cockroach frass induced airway allergic inflammation [15]. However, crude cockroach extract may not be the optimum antigen representing cockroach allergen potency. We have thus performed the profiling of N-linked glycans from purified natural Bla g 2 by MALDI-MS. We, for the first time, demonstrate that Bla g 2 contains a significant amount of small, mannose-terminated glycans with and without fucose (e.g., Man3GlcNAc2, Man3GlcNAc3, and Man3Fuc2GlcNAc2). This finding is important, as it may reveal the distinguishing structural features on Bla g 2 which facilitate recognition of the allergens by the innate immune system. Moreover, glycosylation is the key feature of many allergens [26], and mannose seems to be the dominant sugar moiety associated with some environmental allergens [47], [48]. Furthermore, by comparing with mammalian glycosolation, the processing of N-glycans in insect cells appears to follow a similar initial pathway. However, N-glycans from insect cells are not usually processed to terminally sialylated complex-type structures but are instead modified to oligomannose structures [49], [50]. In addition, when we searched to see whether any of those glycans identified in Bla g 2 from our MALDI-MS analysis have high binding affinity with CD206 in the Functional Glycomics Glycan Database (www.functionalglycomics.org), a database generated by the Consortium for Functional Glycomics (CFG) based on the data generated from the CFG's Scientific Core laboratories and integrated molecule database for glycan-binding proteins, we found that most of those glycans identified in Bla g 2 can bind CD206, suggesting that CD206 may be a major receptor mediating Bla g 2 induced immune responses.

To experimentally determine whether Bla g 2 can directly bind CD206, we have pursued analysis of potential binding of Bla g 2 and soluble CD206, and found a significant binding in a concentration- dependent manner. Interestingly, the binding was significantly inhibited by either CD206 blocking antibody or mannan or mannose. The findings suggest that cockroach allergen contains a natural ligand for CD206 that can bind to CD206.

Recent studies have suggested that the increased number of circulating fibrocytes in asthmatic patients might play an important role in modulation of airway inflammation and tissue remodeling [37] [51], [52]. To determine the role of fibrocytes in cockroach allergen induced allergic immune responses, we isolated and cultured circulating fibrocytes and found that fibrocytes constitutively express CD206 and via CD206 mediate Bla g 2 uptake. CD206 was detected by flow cytometry staining for both surface and intracellular CD206 (with and without permeabilization). Our data indicated that CD206 is expressed weakly at the cell surface, but the detectable levels are increased following membrane permeabilization or increased by intracellular staining. Previous studies have demonstrated that DCs and macrophages via CD206 mediate the uptake of diverse native allergens [28], [29], [30]. Our studies provided additional evidence that fibrocytes via CD206 can mediate Bla g 2 uptake.

Cockroach allergen induced fibrocyte activation was determined by the increase in levels of IL-6 and TNF-α secretion in supernatants of the Bla g 2 treated fibrocytes. Fibrocytes secreted significant amounts of TNF-α and IL-6 when exposed to Bla g 2, and this effect was significantly inhibited by CD206 ligand mannan and mannose pre-treatment, suggesting a possible role of CD206 in mediating Bla g 2-induced fibrocyte activation. Previous studies have shown that mannan can stimulate up-regulation of inflammatory cytokines including interleukin-1β and TNF-α, and differential T helper 1 (Th1)/Th2 cytokines [53], [54]; however, relatively high concentrations (10 to 100 mg/ml) of stimulants are required for activation. Our studies found that neither mannan (2 mg/ml) nor mannose (25 mg/ml) can induce the production of proinflammatory cytokines (TNF-α and IL-6). In contrast, mannan and free mannose can significantly inhibit the increase in Bla g 2 induced proinflammatory cytokines. It is noted, however, that there is only a 30–50% reduction in the secretion of TNF-α and IL-6 after pre-treatment with mannan, suggesting that other receptors (e.g., other CLRs and TLRs) in addition to CD206 in fibrocytes may play a role in cockroach allergen induced immune responses. Further, we cannot rule out the possibility that fibrocyte activation is also mediated through Fc receptors (FcRs) because we and others have found that fibrocytes express two low affinity receptors CD32 (FcγRII) and CD16 (FcγRIII) [32] [55]. Furthermore, it may be argued that allergen-induced inflammatory cytokine secretion in fibrocytes is mediated through the cross-linking of surface-bound, allergen-specific IgE with its high affinity receptor. However, the possibility is unlikely, as in our study fibrocytes did not show detectable levels of the α subunit of the IgE high affinity receptor (FcγRIα) and low affinity receptor (CD23). In this study, we mainly focused on the CD206 mediated cockroach allergen induced fibrocyte activation. As the study progresses, we will investigate whether, in addition to allergen-CD206-axis, FcRs may play a role in cockroach allergen induced fibrocyte activation. Additionally, it is possible that other components in Bla g 2 may play a role in activating fibrocytes given the complexity of allergens.

To delineate the underlying mechanisms of Bla g 2-induced fibrocyte immunity, we have explored signaling events in the Bla g 2-CD206 axis. A recent investigation has demonstrated that CD206 mediated NF-κB activation is critical in innate immune signaling response to pneumocystis [56]. The p38 MAPK pathway has been demonstrated to be involved in the up-regulation of expression of several inflammatory genes (iNOS, IL-8, IL-6) [57]. The ERK-dependent pathway has been reported to be involved in cockroach allergen induced down regulation of TLR-9 and IL12 release [58] and considered essential for the effector functions of CD8^+^ T cells, including Th2 cytokine production and allergic inflammation [59]. Similarly, the JNK pathway has been suggested to be critical in T cell differentiation, T regulatory cell function, and cellular apoptosis signaling [60]. We found that Bla g 2 can induce the activation of NF-κB p65 and three major MAPKs (p38, ERK, and JNK), while Bla g 2-induced activation of p65, ERK, and JNK, but not p38, was inhibited when a non-activating ligand, mannan, was present. Moreover, we found that cockroach allergen induced TNF-α and IL-6 production can be inhibited by the use of inhibitors for p65, p38, and ERK. Interestingly, the JNK inhibitor only showed inhibition for Bla g 2-induced TNF-α, but not IL6. These results suggest that the Bla g 2-CD206 axis involves a complex network of signaling events leading to differential cytokine expression. It is, at present, unclear as to the reason why the activation of Bla g 2-induced MAPK p38 was not inhibited by mannan. This may suggest the existence of an as yet unidentified member of the CLR family mediating Bla g 2′s effect. While the detailed mechanism remains to be fully explored, our current findings may provide an important and novel basis for further investigation of the role of CLRs and fibrocytes in cockroach allergen induced immune responses in asthma.

In summary, we have identified that Bla g 2 contains a significant amount of complex glycans, many of them mannose terminated, and have suggested that the Bla g 2-CD206 axis in fibrocytes may play an important role in cockroach allergen induced allergic immune responses. The fact that blocking CD206 mediated allergen recognition could perhaps prevent the initiation of the subsequent inflammatory cascade opens up the prospect that this pathway could be used for both prophylactic and therapeutic amelioration of allergic diseases like asthma.

## Supporting Information

Table S1 Profile of N-linked glycans from *Bla g2* by MALDI-MS. Putative structures of N-linked glycans shown in the table were assigned by comparison of measured molecular weights of glycans to those of native glycans using Functional Glycomics glycan database (www.functionalglycomics.org) and SimGlycan software (Premier Biosoft, Palo Alto, CA).
(DOC)

Text S1 **Supporting Materials and Methods.**
(DOC)
