# Supplementary material for: Functional Interaction of Cockroach Allergens and Mannose Receptor (CD206) in Human Circulating Fibrocytes
Source: PLoS One. 2013 May 29;8(5):e64105. doi: 10.1371/journal.pone.0064105 (PMC3667076; doi:10.1371/journal.pone.0064105)
Supplement: Table S1 — Profile of N-linked glycans from Bla g2 by MALDI-MS. Putative structures of N-linked glycans shown in the table were assigned by comparison of measured molecular weights of glycans to those of native glycans using Functional Glycomics glycan database (www.functionalglycomics.org) and SimGlycan software (Premier Biosoft, Palo Alto, CA). (DOC) [file pone.0064105.s001.doc]

| **[M+Na]^+^** | **Proposed molecular composition** |
| --- | --- |
| **1141.6** | Man2Fuc1GlcNAc2 |
| **1171.7** | Man3GlcNAc2 |
| **1315.7** | Man2Fuc2GlcNAc2 |
| **1345.7** | Man3Fuc1GlcNAc2 |
| **1375.8** | Man4GlcNAc2 |
| **1416.8** | Man3GlcNAc3 |
| **1519.9** | Man3Fuc2GlcNAc2 |
| **1579.9** | Man5GlcNAc2 |
| **1590.9** | Man3Fuc1GlcNAc3 |
| **1620.9** | Man4GlcNAc3 |
| **1765.0** | Man3Fuc2GlcNAc3 |
| **1784.0** | Man6GlcNAc2 |
| **1866.1** | Man4GlcNAc4 |
| **1907.1** | Man3GlcNAc5 |

**Table S1**. Profile of N-linked glycans from *Bla g2* by MALDI-MS. Putative structures of N-linked glycans shown in the table were assigned by comparison of measured molecular weights of glycans to those of native glycans using Functional Glycomics glycan database (www.functionalglycomics.org) and SimGlycan software (Premier Biosoft, Palo Alto, CA).
