# Supplementary material for: Functional Interaction of Cockroach Allergens and Mannose Receptor (CD206) in Human Circulating Fibrocytes
Source: PLoS One. 2013 May 29;8(5):e64105. doi: 10.1371/journal.pone.0064105 (PMC3667076; doi:10.1371/journal.pone.0064105)
Supplement: Text S1 — Supporting Materials and Methods. (DOC) [file pone.0064105.s002.doc]

**MATERIALS AND METHODS**

**Profiling of** **N-linked glycans from natural purified Bla g2 using mass spectrometry**

1. **Acetone precipitation**

Cold acetone was added to the sample, which was then centrifuged at 4 ^o^C for 15 min and supernatant was removed. Cold acetone was added to the sample again and it was re-centrifuged. The sample was dried down in the speed vacuum.

1. **Release of N-linked glycans from glycopeptide**

The dried sample was dissolved in ammonium bicarbonate buffer (50 mM, pH 8.4), and denatured immediately by heating at 100 °C for 5min prior to trypsin digestion at 37 °C for overnight. After trypsin digestion, the sample was heated at 100 °C for 5 min to deactivate the enzyme. The sample was applied to a C18 Sep-Pak cartridge. Before elution of glycopeptides and peptides, the sample adsorbed in the C18 Sep-Pak cartridge was cleaned with 5% acetic acid. Peptides and glycopeptides then were eluted in series with 20% iso-propanol in 5% acetic acid, 40% iso-propanol in 5% acetic acid and 100% iso-propanol. The propanol fraction was dried, and then sample was treated with a second and third enzyme, peptide N-glycosidase F (New England BioLabs) and peptide N-glycosidase A (Calbiochem) and incubated at 37 °C for 20 hours to release the N-linked glycans. After enzymatic digestion, the sample was passed through a C18 reversed phase cartridge to separate the N-linked glycans from the peptides. The glycan fraction of the sample was eluted with 5% acetic acid and then lyophilized.

1. **Preparation of the per-O-methylated carbohydrates**

The lyophilized carbohydrate fraction was dissolved in dimethylsulfoxide and then methylated with NaOH and methyl iodide. The reaction was quenched by addition of water, and per-O-methylated carbohydrates were extracted with dichloromethane. The organic phase was concentrated to dryness and then the glycans were passed through a C18 Sep-Pak, eluted with 85 % acetonitrile, dried under a stream of nitrogen, and dissolved in methanol prior to analysis by mass spectrometry.

1. **Matrix-assisted laser-desorption ionization time-of-flight mass spectrometry (MALDI/TOF-MS).**

Profiling of N-linked glycans was performed initially using MALDI/TOF-MS on a 4700 Proteomics analyzer (Applied Biosystems). Permethylated glycans (~1 µL) were crystallized on a MALDI plate with 1 µL of 2, 3-dihydroxybenzoic acid (DHBA, 20 mg/mL solution in 50 % methanol) as matrix. All spectra were acquired in the reflector positive ion mode and we averaged the spectra of 50 laser shots.

1. **Assignment of putative glycan structure**

Glycan structures were assigned by comparison of measured m/z values with m/z values calculated based on putative composition of native glycans using the Functional Glycomics glycan database (www.functionalglycomics.org) and SimGlycan software (Premier Biosoft, Palo Alto, CA).
